# Prevalence and severity of work-related musculoskeletal disorders among military firefighters in Manaus: a cross-sectional study*

**DOI:** 10.1590/1980-220X-REEUSP-2025-0205en

**Published:** 2026-10-05

**Authors:** Clodoaldo da Silva Almeida, Ana Lúcia Siqueira Costa Calache, David Lopes, Ednário Barbosa de Mendonça

**Affiliations:** 1 Universidade de São Paulo, Escola de Enfermagem, Programa Saúde do Adulto, São Paulo, SP, Brazil.; 2 Universidade Federal do Amazonas, Escola de Enfermagem de Manaus, Manaus, AM, Brazil.; 3 Universidade Federal de Campina Grande, Campina Grande, PB, Brazil.

**Keywords:** Cumulative Trauma Disorders, Firefighters, Occupational Health, Ergonomics, Nursing

## Abstract

**Objective:**

To assess the prevalence and severity of work-related musculoskeletal disorders (WRMDs) among military firefighters in Manaus.

**Method:**

A cross-sectional study involving 272 active firefighters (79.3% of the workforce of the Capital Fire Department/CBMAM). A sociodemographic/occupational questionnaire and the Nordic Musculoskeletal Symptoms Questionnaire (NMQ, Brazilian version) were used. Data were collected via REDCap (March 2024). Analyses were conducted in RStudio using nonparametric tests (Mann-Whitney, Kruskal-Wallis, Spearman; α = 5%) and effect sizes (95% CI).

**Results:**

The sample was predominantly male (76.8%), with a mean age of 42.5 years; most participants were police officers (79.8%) and engaged in operational duties (84.2%). High occupational exposure was observed (shift work: 74.6%; overtime: 93.0%) as well as regular physical activity (87.9%). The lumbar region showed the highest prevalences of pain, work absenteeism, and seeking medical care. The severity of WRMDs varied across units (p = 0.010; η^2^ = 0.045), with higher values in COBOM and the 1st BI. Correlations between the severity index and pain over 12 months, pain over 7 days, work disability, and healthcare utilization reflected the instrument’s internal consistency.

**Conclusion:**

Military firefighters presented a high prevalence and severity of work-related musculoskeletal disorders (WRMDs), with variations across units. The findings support initiatives in health surveillance, ergonomics, and work organization, especially within the field of occupational nursing.

## INTRODUCTION

The type of work performed by military firefighters is recognized as one of the most physically and psychosocially demanding occupations, characterized by simultaneous exposure to physical, chemical, biological, ergonomic, and organizational risks inherent in rescue operations, firefighting, and prehospital care^(1,2,3)^. This set of demands places this occupational group among those most vulnerable to Work-Related Musculoskeletal Disorders (WRMDs), conditions that rank among the leading causes of sick leave, temporary disability, and early retirement in military fire departments worldwide^(4,5)^.

National and international studies have demonstrated high prevalences of WRMDs among firefighters, often ranging from 50% to 80% over 12-month periods, with the highest incidence in areas such as the lumbar spine, shoulders, knees, and neck^(4,5,6,7)^. These patterns reflect the biomechanical demands of the job, including lifting and carrying loads, forced postures, prolonged use of personal protective equipment, and moving victims^(6,8,9)^. In addition, factors such as length of service, shift schedules, long workdays, insufficient physical activity, and organizational characteristics of the job have been associated with the occurrence and severity of WRMDs, with documented impacts on functional capacity, absenteeism, and quality of life^(2,3,7,9)^. In summary, the literature demonstrates that firefighters constitute a population with a high burden of musculoskeletal symptoms, influenced by intense physical demands and specific organizational conditions.

Despite this body of evidence, some questions remain unanswered, such as the characterization of WRMDs in specific regional contexts, especially in military fire departments in the Amazon region. These departments face unique operational challenges—extensive logistical distances, adverse weather conditions, high-volume river-related incidents, inhospitable forest environments, and structural support limitations—which can alter both occupational exposure and the profile of musculoskeletal disorders^(10,11)^. Added to this is the scarcity of Brazilian studies that link the prevalence, severity, and anatomical distribution of WRMDs with direct applicability to occupational nursing, particularly in the North region^(12)^.

This gap limits the planning of surveillance and prevention measures that are sensitive to the institutional and territorial realities of these organizations, leaving open the question of whether the profile of musculoskeletal disorders observed in other regions is replicated in this setting, which is characterized by specific climatic, logistical, and healthcare conditions. Given this situation, generating local evidence is a necessary step to inform monitoring, prevention, and care strategies consistent with the specific characteristics of the population served—the direct field of action of occupational nursing. Thus, the present study aimed to assess the prevalence and severity of work-related musculoskeletal disorders among military firefighters in Manaus.

## METHOD

### Study Type and Location

This was an observational, cross-sectional, analytical study conducted in March 2024 at the Capital Fire Department (CBC) of the Amazonas Military Fire Department (CBMAM). The study was conducted in accordance with the guidelines of the *Strengthening the Reporting of Observational Studies in Epidemiology – STROBE* initiative^(13)^.

### Population and Sample

The study included 343 military firefighters, selected through non-probabilistic convenience sampling. The final number of participants (n = 272) resulted from the feasibility of access and the voluntary participation of the firefighters during the data collection period, which corresponds to approximately 79.3% of the CBC’s workforce, as shown in Figure 1.

**Figure 1:**
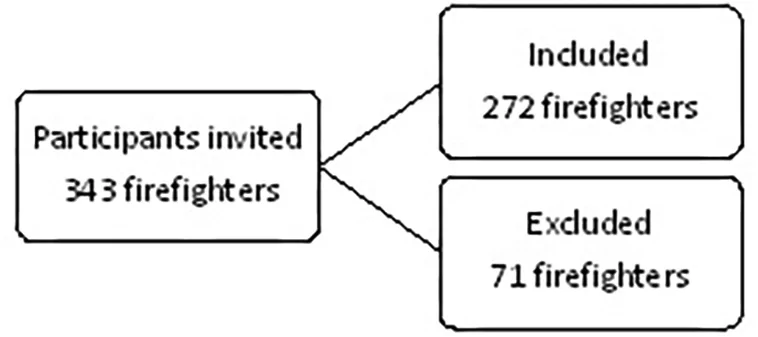
Sample flowchart of study participants – CBC/CBMAM, Manaus, AM, Brazil, 2024.

**Source:** Research form (2024).

The study included active-duty military firefighters stationed in Manaus, in the state of Amazonas, Brazil. Those who, during the data collection period, were on vacation, on leave (medical, bonus, or special), in training, on leave for any reason, inactive, had withdrawn from the study, or had provided incomplete responses were excluded.

### Sample Size Calculation

Since this was a non-probabilistic convenience sample, no formal sample size calculation was performed. The final number of participants (n = 272) resulted from the feasibility of accessing active-duty firefighters, representing 79.3% of the CBC’s workforce—a figure considered sufficient for the purposes of the study. It should be noted that, due to this study design, the findings are not intended to draw inferences about the population as a whole, but rather to describe the profile and identify associations within the study group.

### Data Collection

Data collection took place in March 2024 via a link sent through the fire departments’ WhatsApp groups. After accepting the invitation and signing the informed consent form (ICF), participants completed the questionnaires using the *Research Electronic Data Capture (REDCap)* software from *Vanderbilt University*, which ensured the secure online collection and management of data. The process included five stages: project presentation, preliminary testing, sending invitations, data collection and storage, and final export for statistical analysis, in compliance with the General Data Protection Law (LGPD)^(14)^.

### Instruments Used

The Nordic Musculoskeletal Questionnaire, NMQ was used in its version validated for use in Brazil^(15)^ to assess the prevalence and severity of musculoskeletal symptoms, referred to in this study as Work-Related Musculoskeletal Disorders (WRMDs).

The NMQ assigns a severity index to each anatomical region, ranging from 0 to 4, where 0 indicates the absence of symptoms; 1, symptoms in the past 12 months or in the previous 7 days; 2, symptoms in both periods; 3, presence of symptoms with absence from work; and 4, prolonged absence from work associated with symptoms in both periods. Additionally, the instrument assesses the functional impact of pain, including impairment in daily activities, a variable used in the correlation analyses.

The severity index was calculated based on the NMQ scores for each anatomical region (0 to 4), where higher values indicate greater symptom persistence and greater functional impact. For each participant, the regional scores were averaged, yielding a standardized measure ranging from 0 to 4 that is comparable across individuals and cohorts. Since the index results from a combination of clinical variables—pain, impairment of activities, and healthcare-seeking behavior—correlations with these same components reflect its internal structure, reducing the statistical independence of the associations. Thus, these correlations should be interpreted with caution, as they reflect the instrument’s internal consistency^(15)^.

### Data Processing and Analysis

The data were exported as CSV files, processed in Excel, and analyzed in RStudio (version 2024.12.1). Descriptive analyses included frequencies, means, standard deviations, and medians. The severity index was evaluated according to its ordinal scale, and nonparametric statistical tests—such as the Mann-Whitney, Kruskal-Wallis, and Spearman’s correlation tests—were applied to identify significant associations, with a significance level of 5%. In addition to the significance tests, effect size measures were calculated to estimate the magnitude of the associations: eta-squared (η^2^) for the Kruskal–Wallis test, Cliff’s delta (δ) for comparisons between two groups (Mann–Whitney), and Spearman’s correlation coefficient (ρ) with a 95% confidence interval (95% CI) for correlations. This approach complements the statistical analysis, highlighting not only the statistical significance but also the practical relevance of the results.

It should be noted that Cliff’s delta (δ) is used to compare two groups; values close to 0.1 indicate a small effect, 0.3 a medium effect, and greater than 0.5 a large effect. Eta-squared (η^2^) is used in comparisons among three or more groups (Kruskal–Wallis), with reference values of 0.01 = small, 0.06 = medium, and 0.14 = large; furthermore, Spearman’s correlation (ρ) assesses monotonic correlations, with interpretive ranges of 0.1 to 0.3 for weak correlations, 0.31 to 0.5 for moderate correlations, and above 0.5 for strong correlations. No imputation procedure was performed for missing data, since participants with incomplete responses were previously excluded from the analytical sample, in accordance with the study’s exclusion criteria.

The statistical analysis included descriptive and inferential procedures, with categorical variables presented as absolute and relative frequencies and continuous variables as measures of central tendency and dispersion, according to the observed distribution. To compare medians between groups, the Mann-Whitney and Kruskal-Wallis tests were used, given the nonparametric nature of the severity variable. Correlations between the severity index and the other variables were estimated using Spearman’s correlation coefficient and interpreted according to the conventional classification of magnitude.

It is recognized that some of the variables correlated with the severity index—such as pain in the past 12 months, pain in the past 7 days, activity limitations, and seeking medical care—are part of the index’s own composition, establishing a structural rather than independent relationship between distinct constructs. This characteristic was taken into account in the interpretation of the findings, avoiding causal inferences based on these associations. For future studies aiming to estimate independent associations between occupational exposures and musculoskeletal outcomes in cross-sectional designs, we recommend the use of models that directly estimate the prevalence ratio (PR), such as Poisson regression with robust variance or log-binomial models^(16)^, in line with contemporary epidemiological recommendations for prevalence-based outcomes^(17)^ and with methodological discussions regarding the appropriateness of estimators in this type of study design^(18)^.

### Ethical Procedures

The project was cleared by the Human Research Ethics Committee of the Federal University of Amazonas, under opinion No. 6.632.276, and is part of the author’s doctoral dissertation.

## RESULTS

### Sociodemographic and Behavioral Profile

Males predominated among the participants, with the highest concentration in the 40-and-over age group. Regarding lifestyle habits, 43.8% reported alcohol consumption, while smoking was reported in 4.3% of the sample.

### Employment Profile

Most participants (79.8%) were at the rank-and-file level and performed operational duties (84.2%). Regarding length of service, 75.7% had between 1 and 10 years of service at CBMAM, a pattern also observed among those in operational roles (82.7%). Among those in administrative roles, 96.7% had less than 10 years of experience, which may suggest high turnover.

Work demands were intense: 74.6% of participants worked shifts, and 93.0% worked overtime on operational duties. Despite this, 87.9% reported regular physical activity, possibly as a strategy to maintain functional fitness. In terms of staffing, the Rescue and Pre-Hospital Care Unit (GRAPH) (25.0%), the Airfield Firefighting Service (SCI) (20.2%), and the Special Firefighters Battalion (BBE) (18.4%) stood out, highlighting different contexts of exposure to occupational risks.

### Prevalence of Work-Related Musculoskeletal Disorders

The Nordic Questionnaire on Musculoskeletal Symptoms (NMQ) revealed a high prevalence of work-related musculoskeletal disorders (WRMDs), particularly in the lumbar region, which had the highest frequency of pain in the past 12 months (44.1%; 95% CI: 38.1–50.2), as well as the highest percentages of sick leave (23.5%; 95% CI: 18.6–29.0), healthcare-seeking behavior (22.1%; 95% CI: 17.3–27.5), and pain in the past 7 days (24.3%; 95% CI: 19.3–29.8). Also notable were the shoulders, with a 12-month prevalence of pain of 37.5% (95% CI: 31.7–43.5); the upper back, at 35.3% (95% CI: 29.6–41.3); and the knees, at 32.4% (95% CI: 26.8–38.3). These findings suggest significant clinical and functional impact of the symptoms in the study group, as shown in Table 1.

**Table 1:** Prevalence of work-related musculoskeletal disorders by body region and analysis groups, with 95% confidence intervals, among military firefighters in Manaus – AM, Brazil, 2024.

Body region	Pain 12m% (CI95%)	Sick leave 12m% (CI95%)	Office visits 12m% (CI95%)	Pain 7d% (CI95%)
Elbows	11.0% (7.6–15.4)	6.6% (4.0–10.3)	5.5% (3.1–8.9)	6.6% (4.0–10.3)
Knees	32.4% (26.8–38.3)	18.4% (14.0–23.5)	16.9% (12.7–21.9)	19.5% (14.9–24.7)
Shoulders	37.5% (31.7–43.5)	15.8% (11.7–20.7)	16.9% (12.7–21.9)	20.2% (15.6–25.5)
Lower back (lumbar region)	44.1% (38.1–50.2)	23.5% (18.6–29.0)	22.1% (17.3–27.5)	24.3% (19.3–29.8)
Upper back	35.3% (29.6–41.3)	14.3% (10.4–19.1)	16.2% (12.0–21.1)	17.6% (13.3–22.7)
Neck	34.6% (28.9–40.5)	10.3% (7.0–14.5)	13.2% (9.4–17.8)	16.9% (12.7–21.9)
Wrists/Hands	26.1% (21.0–31.7)	11.0% (7.6–15.4)	10.7% (7.3–15.0)	13.6% (9.8–18.3)
Hips/Thighs	26.8% (21.7–32.5)	13.6% (9.8–18.3)	14.0% (10.1–18.7)	13.6% (9.8–18.3)
Ankles/Feet	25.7% (20.6–31.4)	15.1% (11.0–19.9)	12.1% (8.5–16.6)	14.3% (10.4–19.1)

Source: Research form (2024).

### Severity Index for Work-Related Musculoskeletal Disorders

The distribution of the severity index showed a predominance of more severe categories, with a high proportion of cases associated with severe symptoms, sick leave, and prolonged sick leave, indicating an inability to perform activities with potential repercussions on absenteeism and presenteeism. In contrast, mild or moderate levels were less frequent. This pattern reinforces the finding that, when present, symptoms tended to manifest with greater functional impact in the study group.

### Association Between Sociodemographic and Occupational Variables and the Severity Index of Work-Related Musculoskeletal Disorders

In order to examine the relationship between sociodemographic and occupational variables and the WRMD severity index, the study applied nonparametric tests (Mann–Whitney and Kruskal–Wallis), in addition to Spearman’s correlation for age. The corresponding effect sizes were used to estimate the magnitude of the associations.

### Analysis of Statistical Significance

The WRMDs severity index was centered around a median of 3.00, with similar dispersion (IQR = 2.00) across most categories. No significant differences were observed between gender and educational level/job position, and the distributions showed similar quartiles. In contrast, current staffing levels varied across units, with relatively higher values in the 1st BI and COBOM and lower values in the SCI and GRAPH (Table 2).

**Table 2:** Comparison of the severity index of work-related musculoskeletal disorders according to sociodemographic and occupational variables among military firefighters in Manaus – AM, Brazil, 2024.

Variable	Category/batallion	n	Median	Q1	Q3	IQR	p
Sex	Male	209	3.00	1.00	3.00	2.00	0.885^M^
	Female	63	3.00	1.00	3.00	2.00	
Present unit	BIFMA – Forest Fire and Environmental Battalion	30	3.00	1.00	3.00	2.00	0.010* ^K^	
	BBE – Special Firefighting Battalion	50	3.00	1.00	3.00	2.00	
	1st BI – First Fire Battalion	49	3.00	2.00	4.00	2.00	
	SCI – Airfield Firefighting Service	55	3.00	1.00	3.00	2.00	
	GRAPH – Rescue and Pre-Hospital Care Unit	68	3.00	1.00	3.00	2.00	
	COBOM – Military Firefighter Operations Center	20	3.00	2.00	4.00	2.00	
Grade/post	Rank	217	3.00	1.00	3.00	2.00	0.287^M^
	Officer	55	3.00	1.00	3.00	2.00	

Source: Research form (2024).

Caption: (*) Significant difference p < 0.05.

^M^Mann–Whitney test;

^K^Kruskal–Wallis test. Q1 = first quartile; Q3 = third quartile; IQR = interquartile range (Q3–Q1).

### Effect Size and Practical Relevance

Regarding the magnitude of the associations, negligible effects were observed for sex (δ = 0.03) and academic level/rank (δ = 0.08), indicating little or no practical difference in severity between these groups. For current staffing levels, a small effect was found (η^2^ = 0.045), and post hoc analysis indicated greater severity on the COBOM compared to the GRAPH and SCI. Age showed a weak and nonsignificant correlation with the severity index (ρ = 0.12; 95% CI: −0.01 to 0.25), suggesting a limited influence of this factor on the outcome under study (Table 3).

**Table 3:** Association between sociodemographic and occupational variables and the severity index of work-related musculoskeletal disorders among military firefighters in Manaus – AM, Brazil, 2024.

Variable	Category/descriptor	Effect measure	Interpretation	Post-hoc comparisons (Dunn test, Bonferroni correction)
Sex	Men/women	δ = 0.03	Negligible effect	—
Present unit	1st BI – Fire Battalion; BIFMA – Forest Fire and Environment; BBE – Special Firefighters SCI – Airfield Firefighting; GRAPH – Rescue and Pre-Hospital Care; COBOM – Military Firefighter Operations Center	η^2^ = 0.045	Small effect	COBOM > GRAPH, SCI (p < 0.05)
Grade/post	Rank/Officer	δ = 0.08	Negligible effect	—
Age (Spearman correlation)	Age X Severity index	ρ = 0.12 (IC95%: −0.01–0.25)	Weak, non-significant correlation	—

Source: Research form (2024).

Caption: η2 = effect size for the Kruskal–Wallis test; δ = Cliff’s delta for the Mann–Whitney test; ρ = Spearman’s correlation coefficient with a 95% confidence interval (95% CI).

### Internal Consistency of the Severity Index for Work-Related Musculoskeletal Disorders

In order to assess the internal consistency of the severity index, we examined the strength of the association between the composite score and the clinical and functional indicators that make up its structure. Spearman’s correlation coefficients were high for pain in the past 12 months (ρ = 0.896; 95% CI: 0.870–0.917), pain in the past 7 days (ρ = 0.775; 95% CI: 0.723–0.819), impairment in usual activities (ρ = 0.767; 95% CI: 0.713–0.812), and seeking healthcare (ρ = 0.695; 95% CI: 0.628–0.752), with p < 0.001 in all tests (Table 4).

**Table 4:** Internal consistency coefficients between the components of the Nordic Questionnaire on Musculoskeletal Symptoms (NQMS) and the DORT severity index among military firefighters in Manaus – AM, Brazil, 2024.

Variable	Test	Measure of effect (*)	Value	p-value	Interpretation	CI95%
Pain in the past 12 months × severity index	Spearman	ρ	0.896	<0.001	high internal consistency	0.870 a 0.917
Pain in the past 7 days × severity index			0.775	<0.001	high internal consistency	0.723 a 0.819
Limitation of activities × severity index			0.767	<0.001	high internal consistency	0.713 a 0.812
Seeking medical care × severity index			0.695	<0.001	high internal consistency	0.628 a 0.752

Source: Research form (2024).

Caption: ρ = Spearman’s correlation coefficient; 95% CI = 95% confidence interval. Note: The coefficients presented reflect the structural consistency between the instrument’s components and the composite score (severity index) and do not constitute independent associations, given that the same variables are included in the composition of the index.

These results should not be interpreted as independent associations, but rather as an expression of the construct’s internal consistency, since the indicators analyzed are part of the index’s composition itself. The observed magnitude confirms that the score coherently synthesizes the dimensions—temporal (presence of symptoms across different time windows), functional (impairment of activities), and care-related (seeking care)—proposed by the NMQ, reinforcing its suitability as a summary measure of DORT severity in the present sample. Interpreting these coefficients as evidence of a causal or predictive association between clinical variables and severity would therefore be methodologically inappropriate.

## DISCUSSION

The findings of this study reflect the operational reality of military firefighters in the Amazon region, which is characterized by intense physical demands, adverse environmental conditions—including exposure to air pollutants from wildfires— and logistical challenges that shape their daily work^(19)^. Although these characteristics were not directly measured, they constitute the occupational setting in which the participants work and help contextualize the high prevalence of musculoskeletal symptoms observed. In this sense, the study contributes by providing local evidence on a condition that remains poorly described among military firefighters in the Northern region.

From a sociodemographic perspective, a male predominance and a higher concentration of participants aged 40 or older were observed, a pattern similar to that described in national and international surveys^(20,21,22)^. This profile may reflect both the historical composition of the fire departments and a possible aging of the workforce, a phenomenon already documented in emergency services in other countries^(23)^. The higher proportion of female participants compared to previous studies in the Amazon suggests gradual changes in recruitment profiles, although the proportion remains low.

In the workplace, the predominant profile was that of rank-and-file firefighters in operational roles, a pattern consistent with fire departments in different countries and with characterizations of the profession in the Brazilian context^(24)^. Work schedules characterized by shift work and frequent overtime may be associated with greater physical and mental exhaustion, especially when rest periods are insufficient, as described in other occupational contexts^(25,26,27)^. Although the cross-sectional design does not allow for establishing direction or causality, such conditions create a work environment potentially conducive to the accumulation of physical and psychosocial demands.

The high frequency of musculoskeletal symptoms, especially in areas such as the lumbar spine, shoulders, upper back, neck, and knees, is consistent with the profile of physical demands inherent to the military firefighter’s work^(26,27,28)^. These patterns can be partially explained by biomechanical mechanisms widely described in the literature, such as lifting and carrying loads, rapid movements, forced postures, prolonged use of personal protective equipment, and moving victims in unstable environments. In the Amazonian context, additional factors—such as river operations, long travel distances, extreme heat, and structural limitations—may intensify physical demands and reduce effective recovery time between shifts, contributing to the longer persistence of symptoms.

The association between WRMD severity and current staffing levels, although of small magnitude, suggests that organizational and operational differences between units may influence the distribution of symptoms. The higher medians observed at COBOM and the 1st BI indicate that certain work contexts may be related to a higher burden of symptoms, although it is not possible to infer causality. On the other hand, the negligible or weak associations with gender, hierarchical level, and age suggest that, in this sample, symptom severity was less related to individual characteristics and more closely linked to the occupational context and the clinical-functional components captured by the instrument—a finding supported by some of the literature^(29,30)^.

The high correlations between the severity index and variables such as pain in the past 12 months, pain in the past 7 days, activity limitations, and seeking medical care should be interpreted as an expression of the instrument’s internal consistency, rather than as independent associations. These indicators are integral to the index’s composition itself, so the observed magnitude reflects the construct’s structural coherence rather than causal or predictive relationships between distinct variables.

This study has limitations that should be considered. The convenience sample limits the generalizability of the findings to the entire organization. The use of self-report data may introduce memory or perceptual biases, especially regarding the intensity and duration of symptoms. Furthermore, although bivariate associations were explored, multivariate analyses could provide more robust adjusted estimates, reducing potential confounders. Finally, the cross-sectional design prevents establishing temporal relationships or inferring causality between occupational exposures and symptom severity.

Furthermore, although the application of multivariate models—such as Poisson regression with robust variance—could provide adjusted prevalence ratios, we chose to restrict the analysis to the bivariate level, consistent with the descriptive-exploratory design and convenience sampling adopted. This choice aims to preserve inferential transparency and avoid attributing statistical precision that is incompatible with the study design. The findings presented here should therefore be interpreted as indicative of an association, rather than as adjusted effect estimates generalizable to the population of military firefighters.

Despite these limitations, the study provides relevant evidence for occupational nursing by characterizing the prevalence and severity of WRMDs in a largely unexplored regional context, reinforcing the need for health surveillance strategies, ergonomic interventions, and organizational measures that are sensitive to the specific characteristics of different units. It is recommended that future studies, using a longitudinal design or probabilistic sampling, apply multivariate models—such as Poisson regression with robust variance—to estimate adjusted prevalence ratios and explore interactions between workload, length of service, and physical activity, thereby informing evidence-based preventive programs.

## CONCLUSION

The prevalence and severity of work-related musculoskeletal disorders among military firefighters in Manaus reveal a significant burden of symptoms, especially in body regions critical for operational performance. The differences observed across units suggest that organizational characteristics and the specific demands of each unit may influence the distribution of symptoms, although it is not possible to establish causality.

The findings reinforce the need for systematic health surveillance, ergonomic interventions, and organizational strategies that take into account the specific characteristics of the Amazonian context. For occupational nursing, the results provide a basis for planning preventive, educational, and care related practices aimed at reducing the musculoskeletal burden and promoting the functional health of these professionals. Future studies with analytical designs and adjusted models may deepen our understanding of the factors associated with WRMDs in this population.

## Data Availability

The entire dataset supporting the results of this study is available upon request to the corresponding author.
