# Supplementary material for: Prevalence and severity of work-related musculoskeletal disorders among military firefighters in Manaus: a cross-sectional study
Source: Rev Esc Enferm USP. 2026 Oct 5;60:e20250205. doi: 10.1590/1980-220X-REEUSP-2025-0205en (PMC13648200; doi:10.1590/1980-220X-REEUSP-2025-0205en)
Supplement: Translation

# Translation: Prevalência e severidade de distúrbios osteomusculares relacionados ao trabalho entre bombeiros militares manauaras: um estudo transversal^*^

**DOI:** 10.1590/1980-220X-REEUSP-2025-0205pt

**Authors:** Clodoaldo da Silva Almeida, Ana Lúcia Siqueira Costa Calache, David Lopes, Ednário Barbosa de Mendonça

**Affiliations:** 1 Universidade de São Paulo, Escola de Enfermagem, Programa Saúde do Adulto, São Paulo, SP, Brasil.; 2 Universidade Federal do Amazonas, Escola de Enfermagem de Manaus, Manaus, AM, Brasil.; 3 Universidade Federal de Campina Grande, Campina Grande, PB, Brasil.

**Keywords:** Transtornos Traumáticos Cumulativos, Bombeiros, Saúde Ocupacional, Ergonomia, Enfermagem

## Abstract

**Objetivo:**

Avaliar a prevalência e a severidade de distúrbios osteomusculares relacionados ao trabalho (DORT) em bombeiros militares de Manaus.

**Método:**

Estudo transversal com 272 bombeiros ativos (79,3% do efetivo do Comando de Bombeiros da Capital/CBMAM). Utilizaram-se questionário sociodemográfico/laboral e o Questionário Nórdico de Sintomas Osteomusculares (QNSO, versão brasileira). A coleta ocorreu via REDCap (março/2024). As análises foram conduzidas no RStudio, com testes não paramétricos (Mann-Whitney, Kruskal-Wallis, Spearman; α = 5%) e medidas de efeito (IC95%).

**Resultados:**

Predominaram homens (76,8%), idade média de 42,5 anos, praças (79,8%) e atividades operacionais (84,2%). Observou-se elevada exposição laboral (turnos: 74,6%; horas extras: 93,0%) e prática regular de atividade física (87,9%). A região lombar apresentou as maiores prevalências de dor, afastamento e procura por atendimento. A severidade dos DORT variou entre lotações (p = 0,010; η^2^ = 0,045), com valores mais elevados no COBOM e no 1.º- BI. As correlações entre o índice de severidade e dor em 12 meses, dor em 7 dias, impedimento e atendimento refletiram consistência interna do instrumento.

**Conclusão:**

Bombeiros militares apresentaram elevada prevalência e severidade de DORT, com variações entre lotações. Os achados subsidiam ações de vigilância em saúde, ergonomia e organização do trabalho, especialmente no âmbito da Enfermagem do Trabalho.

## INTRODUÇÃO

A atividade de bombeiro militar é reconhecida como uma das ocupações mais exigentes do ponto de vista físico e psicossocial, caracterizando-se pela exposição simultânea a riscos físicos, químicos, biológicos, ergonômicos e organizacionais inerentes às operações de resgate, combate a incêndios e atendimento pré-hospitalar^(1,2,3)^. Esse conjunto de demandas coloca a categoria entre os grupos ocupacionais com maior vulnerabilidade aos Distúrbios Osteomusculares Relacionados ao Trabalho (DORT), agravos que figuram entre as principais causas de afastamento, incapacidade temporária e aposentadoria precoce em corporações militares ao redor do mundo^(4,5)^.

Estudos nacionais e internacionais têm demonstrado prevalências elevadas de DORT entre bombeiros, com taxas que variam entre 50% e 80% em períodos de 12 meses. As áreas mais acometidas compreendem as regiões da coluna lombar, ombros, joelhos e pescoço^(4,5,6,7)^. Esses padrões refletem as exigências biomecânicas da atividade, nos quais se incluem levantamento e transporte de cargas, posturas forçadas, uso prolongado de equipamentos de proteção individual e movimentação de vítimas^(6,8,9)^. Além disso, fatores como tempo de serviço, regime de turnos, jornadas prolongadas, prática insuficiente de atividade física e características organizacionais do trabalho têm sido associados à ocorrência e à severidade dos DORT, com impactos documentados sobre capacidade funcional, absenteísmo e qualidade de vida^(2,3,7,9)^. Em síntese, a literatura demonstra que bombeiros constituem uma população com elevada carga de sintomas osteomusculares, influenciada por demandas físicas intensas e por condições organizacionais específicas.

Apesar desse corpo de evidências, algumas questões permanecem em aberto, como a caracterização dos DORT em contextos regionais específicos, especialmente nas corporações bombeiro-militar da região amazônica. Essas corporações enfrentam particularidades operacionais — grandes distâncias logísticas, condições climáticas adversas, ocorrências fluviais de alto volume, ambientes florestais inóspitos e limitações estruturais de suporte — que podem modificar tanto a exposição ocupacional quanto o perfil de adoecimento osteomuscular^(10,11)^. Soma-se a isso a escassez de estudos brasileiros que articulem prevalência, severidade e distribuição anatômica dos DORT com aplicabilidade direta à Enfermagem do Trabalho, sobretudo na região Norte^(12)^.

Essa lacuna limita o planejamento de ações de vigilância e prevenção sensíveis às realidades institucional e territorial dessas corporações, permanecendo em aberto saber se o perfil de adoecimento osteomuscular observado em outras regiões se reproduz nesse cenário, marcado por particularidades climáticas, logísticas e assistenciais. Diante desse quadro, produzir evidências locais constitui passo necessário para subsidiar estratégias de monitoramento, prevenção e cuidado coerentes com as especificidades da população assistida — campo de atuação direta da Enfermagem do Trabalho. Assim, o presente estudo teve como objetivo avaliar a prevalência e a severidade dos distúrbios osteomusculares relacionados ao trabalho em bombeiros militares de Manaus.

## MÉTODO

### Tipo e Local do Estudo

Estudo observacional, transversal, analítico, realizado em março de 2024, no Comando de Bombeiros da Capital (CBC) do Corpo de Bombeiros Militar do Amazonas (CBMAM). O estudo foi feito com base nas diretrizes da iniciativa *Strengthening the Reporting of Observational Studies in Epidemiology – STROBE* ^(13)^.

### População e Amostra

O estudo contou com a participação de 343 bombeiros militares, selecionados por amostragem não probabilística do tipo conveniência. O número final de participantes (n = 272) resultou da viabilidade de acesso e da adesão voluntária dos bombeiros durante o período de coleta de dados, o que corresponde a aproximadamente 79,3% do efetivo do CBC, conforme demonstrado na Figura 1.

**Figura 1:** Fluxo amostral dos participantes do estudo – CBC/CBMAM, Manaus AM, Brasil, 2024.

**Fonte:** Questionário da pesquisa (2024).

Foram incluídos bombeiros militares em serviço ativo, lotados em Manaus, no estado do Amazonas, no Brasil. Foram excluídos aqueles que, no período da coleta, estavam de férias, em licença (saúde, prêmio ou especial), em treinamento, afastados por qualquer motivo, inativos, desistentes ou com respostas incompletas.

### Cálculo Amostral

Por se tratar de amostragem não probabilística por conveniência, não foi realizado cálculo amostral formal. O número final de participantes (n = 272) decorreu da viabilidade de acesso aos bombeiros em serviço ativo, o que representa 79,3% do efetivo do CBC, valor considerado suficiente para os propósitos da pesquisa. Ressalta-se que, por escolha desse delineamento, os achados não visam à inferência populacional, mas sim à descrição do perfil e à identificação de associações no grupo estudado.

### Coleta de Dados

A coleta ocorreu em março de 2024 via link enviado pelo WhatsApp dos batalhões. Após aceitar o convite e assinar o termo de consentimento livre e esclarecido (TCLE), os participantes responderam aos instrumentos no software *Research Electronic Data Capture (REDCap)*, da *Vanderbilt University*, que assegurou a coleta e a gestão seguras dos dados online. O processo incluiu cinco etapas: apresentação do projeto, testes preliminares, envio de convites, coleta e armazenamento dos dados e exportação final para análise estatística, em conformidade com a Lei Geral de Proteção de Dados (LGPD)^(14)^.

### Instrumentos Utilizados

Utilizou-se o Questionário Nórdico de Sintomas Osteomusculares (QNSO), em sua versão validada para uso no Brasil^(15)^, empregada na avaliação da prevalência e da severidade de sintomas osteomusculares, neste estudo denominados Distúrbios Osteomusculares Relacionados ao Trabalho (DORT).

O QNSO atribui um índice de severidade a cada região anatômica, variando de 0 a 4, em que 0 indica ausência de sintomas; 1, sintomas nos últimos 12 meses ou nos 7 dias anteriores; 2, sintomas em ambos os períodos; 3, presença de sintomas com afastamento das atividades; e 4, afastamento prolongado associado a sintomas em ambos os períodos. Adicionalmente, o instrumento investiga repercussões funcionais da dor, incluindo o impedimento de atividades habituais, variável empregada nas análises de correlação.

O índice de severidade foi calculado a partir dos escores do QNSO para cada região anatômica (0 a 4), em que valores maiores indicam maior persistência dos sintomas e maior repercussão funcional. Para cada participante, obteve-se a média dos escores regionais e a produção de gerando uma medida padronizada de 0 a 4, comparável entre indivíduos e lotações. Como o índice resulta da combinação de variáveis clínicas — dor, impedimento de atividades e procura por atendimento — as correlações com esses mesmos componentes refletem sua estrutura interna, com redução da independência estatística das associações. Assim, tais correlações devem ser interpretadas com cautela, pois expressam consistência interna do instrumento^(15)^.

### Tratamento e Análise dos Dados

Os dados foram exportados em CSV, tratados no Excel e analisados no RStudio (versão 2024.12.1). As análises descritivas incluíram frequências, médias, desvios-padrão e medianas. O índice de severidade foi avaliado conforme sua estrutura ordinal, e testes estatísticos não paramétricos, como Mann-Whitney, Kruskal-Wallis e correlação de Spearman, foram aplicados para verificar associações significativas, com nível de significância de 5%. Além dos testes de significância, foram calculadas medidas de tamanho de efeito para estimar a magnitude das associações: o eta quadrado (η^2^) para o teste de Kruskal–Wallis, o delta de Cliff (δ) para comparações entre dois grupos (Mann–Whitney) e o coeficiente de Spearman (ρ) com intervalo de confiança de 95% (IC95%) para correlações. Essa abordagem complementa a análise estatística, evidenciando não apenas a significância, mas também a relevância prática dos resultados.

Ressalta-se que a medida de efeito Delta de Cliff (δ) é usada para comparar dois grupos; valores próximos de 0,1 indicam efeito pequeno, 0,3 médio, e maior que 0,5, efeito grande. O eta quadrado (η^2^) é usado em comparações entre três ou mais grupos (Kruskal–Wallis) cujos valores de referência são 0,01 = pequeno, 0,06 = médio, 0,14 = grande; ainda, a correlação de Spearman (ρ) avalia correlações monotônicas, cujo resultado interpretativo é de 0,1 a 0,3 para correlações fracas, 0,31 a 0,5 para correlações moderadas e acima de 0,5 para correlações fortes. Não foi realizado procedimento de imputação de dados ausentes, uma vez que os participantes com respostas incompletas foram previamente excluídos da amostra analítica, conforme os critérios de exclusão do estudo.

A análise estatística contemplou procedimentos descritivos e inferenciais, com apresentação das variáveis categóricas em frequências absolutas e relativas e das variáveis contínuas em medidas de tendência central e dispersão, conforme a distribuição observada. Para a comparação de medianas entre grupos, utilizaram-se os testes de Mann-Whitney e Kruskal-Wallis, tendo em vista a natureza não paramétrica da variável de severidade. As correlações entre o índice de severidade e as demais variáveis foram estimadas pelo coeficiente de Spearman, interpretadas conforme classificação convencional de magnitude.

Reconhece-se que, parte das variáveis correlacionadas com o índice de severidade — como dor nos últimos 12 meses, dor nos últimos 7 dias, impedimento de atividades e procura por atendimento — integram a própria composição do índice e configuram a relação de natureza estrutural e não independente entre construtos distintos. Tal característica foi considerada na interpretação dos achados, onde evitou-se inferências causais a partir dessas associações. Para investigações futuras que pretendam estimar associações independentes entre exposições ocupacionais e desfechos osteomusculares em delineamentos transversais, recomenda-se o emprego de modelos que estimem diretamente a razão de prevalência (RP), como a regressão de Poisson com variância robusta ou modelos log-binomiais^(16)^, em consonância com as recomendações da epidemiologia contemporânea para desfechos prevalentes^(17)^ e com as discussões metodológicas sobre adequação de estimadores nesse tipo de delineamento^(18)^.

### Procedimentos Éticos

O projeto foi aprovado pelo Comitê de Ética em Pesquisa com Seres Humanos da Universidade Federal do Amazonas, sob o parecer nº- 6.632.276, e integra a tese de doutoramento do autor.

## RESULTADOS

### Perfil Sociodemográfico e Comportamental

Entre os participantes, predominou o sexo masculino, com maior concentração na faixa etária de 40 anos ou mais. Quanto aos hábitos de vida, 43,8% relataram consumo de bebidas alcoólicas, enquanto o tabagismo foi registrado em 4,3% da amostra.

### Perfil Laboral

A maioria dos participantes (79,8%) pertencia ao nível hierárquico de praças e desempenhava atividades operacionais (84,2%). Quanto ao tempo de serviço, 75,7% tinha entre 1 e 10 anos de atuação no CBMAM, padrão também observado entre os que exerciam função operacional (82,7%). Entre aqueles em atividade administrativa, 96,7% tinham menos de 10 anos de experiência, o que pode sugerir rotatividade.

As demandas laborais mostraram-se intensas: 74,6% dos participantes trabalhavam por turnos e 93,0% realizavam horas extras em atividades operacionais. Apesar disso, 87,9% referiram prática regular de atividade física, possivelmente como estratégia de manutenção da aptidão funcional. Em relação à lotação, destacaram-se o Grupamento de Resgate e Atendimento Pré-Hospitalar (GRAPH) (25,0%), o Serviço de Combate a Incêndio em Aeródromo (SCI) (20,2%) e o Batalhão de Bombeiros Especiais (BBE) (18,4%), o que evidencia diferentes contextos de exposição a riscos ocupacionais.

### Prevalência dos Distúrbios Osteomusculares Relacionados ao Trabalho

O Questionário Nórdico de Sintomas Osteomusculares (QNSO) evidenciou alta prevalência dos DORT, com destaque para a região lombar, que apresentou a maior frequência de dor nos últimos 12 meses (44,1%; IC95%: 38,1–50,2), além dos maiores percentuais de afastamento (23,5%; IC95%: 18,6–29,0), procura por atendimento (22,1%; IC95%: 17,3–27,5) e dor nos últimos 7 dias (24,3%; IC95%: 19,3–29,8). Também se destacaram os ombros, com prevalência de dor em 12 meses de 37,5% (IC95%: 31,7–43,5), a parte superior das costas, com 35,3% (IC95%: 29,6–41,3), e os joelhos, com 32,4% (IC95%: 26,8–38,3). Esses achados sugerem importante repercussão clínica e funcional dos sintomas no grupo estudado, conforme demonstrado na Tabela 1.

**Tabela 1:** Prevalência de distúrbios osteomusculares relacionados ao trabalho por região corporal e blocos de análise, com intervalos de confiança de 95% entre bombeiros militares de Manaus – AM, Brasil, 2024.

Região corporal	Dor 12m% (IC95%)	Afastamento 12m% (IC95%)	Consultas 12m% (IC95%)	Dor 7d% (IC95%)
Cotovelos	11,0% (7,6–15,4)	6,6% (4,0–10,3)	5,5% (3,1–8,9)	6,6% (4,0–10,3)
Joelhos	32,4% (26,8–38,3)	18,4% (14,0–23,5)	16,9% (12,7–21,9)	19,5% (14,9–24,7)
Ombros	37,5% (31,7–43,5)	15,8% (11,7–20,7)	16,9% (12,7–21,9)	20,2% (15,6–25,5)
Parte inferior das costas (lombar)	44,1% (38,1–50,2)	23,5% (18,6–29,0)	22,1% (17,3–27,5)	24,3% (19,3–29,8)
Parte superior das costas	35,3% (29,6–41,3)	14,3% (10,4–19,1)	16,2% (12,0–21,1)	17,6% (13,3–22,7)
Pescoço	34,6% (28,9–40,5)	10,3% (7,0–14,5)	13,2% (9,4–17,8)	16,9% (12,7–21,9)
Punhos/Mãos	26,1% (21,0–31,7)	11,0% (7,6–15,4)	10,7% (7,3–15,0)	13,6% (9,8–18,3)
Quadris/Coxas	26,8% (21,7–32,5)	13,6% (9,8–18,3)	14,0% (10,1–18,7)	13,6% (9,8–18,3)
Tornozelos/Pés	25,7% (20,6–31,4)	15,1% (11,0–19,9)	12,1% (8,5–16,6)	14,3% (10,4–19,1)

Fonte: Questionário da pesquisa (2024).

### Índice de Severidade de Distúrbios Osteomusculares Relacionados ao Trabalho

A distribuição do índice de severidade apontou predominância de categorias de maior gravidade, com elevada proporção de registros associados a sintomas graves, afastamento e afastamento prolongado, cujo significado indica impedimento de atividades com potencial repercussão no absenteísmo e no presenteísmo. Em contraste, os níveis leves ou moderados foram menos frequentes. Esse padrão reforça que, quando presentes, os sintomas tendiam a se manifestar com maior repercussão funcional no grupo estudado.

### Associação Entre Variáveis Sociodemográficas, Laborais e o Índice de Severidade dos Distúrbios Osteomusculares Relacionados ao Trabalho

Para examinar a relação entre variáveis sociodemográficas e laborais e o índice de severidade dos DORT, foram aplicados testes não paramétricos (Mann–Whitney e Kruskal–Wallis), além de correlação de Spearman para a idade. As medidas de efeito correspondentes foram utilizadas para estimar a magnitude das associações.

### Análise de Significância Estatística

O índice de severidade dos DORT apresentou padrão centrado na mediana de 3,00, com dispersão semelhante (IQR = 2,00) na maioria das categorias. Não foram observadas diferenças relevantes entre sexo e graduação/posto, e as distribuições apresentaram quartis semelhantes. Em contrapartida, a lotação atual mostrou variação entre unidades, com valores relativamente mais elevados no 1.º BI e no COBOM e distribuição mais baixa no SCI e no GRAPH (Tabela 2).

**Tabela 2:** Comparação do índice de severidade dos distúrbios osteomusculares relacionados ao trabalho, segundo variáveis sociodemográficas e laborais entre bombeiros militares de Manaus – AM, Brasil, 2024.

Variável	Categoria/batalhão	n	Mediana	Q1	Q3	IQR	p
Sexo	Masculino	209	3,00	1,00	3,00	2,00	0,885^M^
	Feminino	63	3,00	1,00	3,00	2,00	
Lotação atual	BIFMA – Batalhão de Incêndio Florestal e Meio Ambiente	30	3,00	1,00	3,00	2,00	0,010* ^K^
	BBE – Batalhão de Bombeiros Especiais	50	3,00	1,00	3,00	2,00	
	1.º- BI – Primeiro Batalhão de Incêndio	49	3,00	2,00	4,00	2,00	
	SCI – Serviço de Combate a Incêndio em Aeródromo	55	3,00	1,00	3,00	2,00	
	GRAPH – Grupamento de Resgate e Atendimento Pré-Hospitalar	68	3,00	1,00	3,00	2,00	
	COBOM – Centro de Operações Bombeiro Militar	20	3,00	2,00	4,00	2,00	
Graduação/posto	Praça	217	3,00	1,00	3,00	2,00	0,287^M^
	Oficial	55	3,00	1,00	3,00	2,00	

Fonte: Questionário da pesquisa (2024).

Legenda: (*) Diferença significativa p < 0,05.

^M^Teste de Mann–Whitney;

^K^Teste de Kruskal–Wallis. Q1 = primeiro quartil; Q3 = terceiro quartil; IQR = intervalo interquartil (Q3–Q1).

### Tamanho do Efeito e Relevância Prática

Quanto à magnitude das associações, observaram-se efeitos desprezíveis para sexo (δ = 0,03) e graduação/posto (δ = 0,08), o que reflete em pouca ou nenhuma diferença prática na severidade entre esses grupos. Para a lotação atual, verificou-se um efeito pequeno (η^2^ = 0,045), e a análise pós-hoc indicou maior severidade no COBOM em comparação ao GRAPH e ao SCI. A idade apresentou correlação fraca e não significativa com o índice de severidade (ρ = 0,12; IC95%: −0,01 a 0,25), cujo resultado faz sugerir a influência limitada desse fator sobre o desfecho estudado (Tabela 3).

**Tabela 3:** Associação entre variáveis sociodemográficas e laborais e o índice de severidade dos distúrbios osteomusculares relacionados ao trabalho entre bombeiros militares de Manaus – AM, Brasil, 2024.

Variável	Categoria/descritor	Medida de efeito	Interpretação	Comparações pós-hoc (teste de Dunn, correção Bonferroni)
**Sexo**	Masculino/Feminino	δ = 0,03	Efeito desprezível	—
**Lotação atual**	1.º- BI – Batalhão de Incêndio; BIFMA – Incêndio Florestal e Meio Ambiente; BBE – Bombeiros Especiais; SCI – Combate a Incêndio em Aeródromo; GRAPH – Resgate e Atendimento Pré-Hospitalar; COBOM – Centro de Operações Bombeiro Militar	η^2^ = 0,045	Efeito pequeno	COBOM > GRAPH, SCI (p < 0,05)
**Graduação/posto**	Praça/Oficial	δ = 0,08	Efeito desprezível	—
**Idade (Correlação de Spearman)**	Idade × Índice de Severidade	ρ = 0,12 (IC95%: −0,01 –0,25)	Correlação fraca, não significativa	—

Fonte: Questionário da pesquisa (2024).

Legenda: η^2^ = medida de efeito para o teste de Kruskal–Wallis; δ = delta de Cliff para o teste de Mann–Whitney; ρ = coeficiente de correlação de Spearman com intervalo de confiança de 95% (IC95%).

### Consistência Interna do Índice de Severidade dos Distúrbios Osteomusculares Relacionados ao Trabalho

Para verificar a coerência interna do índice de severidade, examinou-se a magnitude da associação entre o escore composto e os indicadores clínicos e funcionais que compõem sua estrutura. Os coeficientes de Spearman foram elevados para dor nos últimos 12 meses (ρ = 0,896; IC95%: 0,870–0,917), dor nos últimos 7 dias (ρ = 0,775; IC95%: 0,723–0,819), impedimento de atividades habituais (ρ = 0,767; IC95%: 0,713–0,812) e procura por atendimento de saúde (ρ = 0,695; IC95%: 0,628–0,752), com p < 0,001 em todos os testes (Tabela 4).

**Tabela 4:** Coeficientes de consistência interna entre os componentes do Questionário Nórdico de Sintomas Osteomusculares (QNSO) e o índice de severidade dos DORT, entre bombeiros militares de Manaus – AM, Brasil, 2024.

Variável	Teste	Medida de efeito (*)	Valor	p-valor	Interpretação	IC95%
Dor nos últimos 12 meses × índice de severidade	Spearman	ρ	0,896	<0,001	consistência interna elevada	0,870 a 0,917
Dor nos últimos 7 dias × índice de severidade			0,775	<0,001	consistência interna elevada	0,723 a 0,819
Impedimento de atividades × índice de severidade			0,767	<0,001	consistência interna elevada	0,713 a 0,812
Procura por atendimento médico × índice de severidade			0,695	<0,001	consistência interna elevada	0,628 a 0,752

Fonte: Questionário de pesquisa (2024).

Legenda: ρ = coeficiente de correlação de Spearman; IC95% = intervalo de confiança de 95%. Nota: Os coeficientes apresentados refletem a coerência estrutural entre os componentes do instrumento e o escore-síntese (índice de severidade), e não constituem associações independentes, dada a inclusão das mesmas variáveis na composição do índice.

Tais resultados não devem ser interpretados como associações independentes, mas como expressão da consistência interna do constructo, uma vez que os indicadores analisados integram a própria composição do índice. A magnitude observada confirma que o escore sintetiza, de forma coerente, as dimensões: temporal (presença de sintomas em diferentes janelas), funcional (impedimento de atividades) e assistencial (procura por cuidado) propostas pelo QNSO, o que reforça sua adequação como medida-resumo da severidade dos DORT na presente amostra. A interpretação destes coeficientes como evidência de associação causal ou preditiva entre variáveis clínicas e severidade seria, portanto, metodologicamente inadequada.

## DISCUSSÃO

Os achados deste estudo dialogam com a realidade operacional dos bombeiros militares da região amazônica, marcada por demandas físicas intensas, condições ambientais adversas — onde se inclui exposição a poluentes atmosféricos provenientes de queimadas — e desafios logísticos que moldam o cotidiano de trabalho^(19)^. Embora tais características não tenham sido mensuradas diretamente, elas compõem o cenário ocupacional no qual os participantes estão inseridos e ajudam a contextualizar a elevada carga de sintomas osteomusculares observada. Nesse sentido, o estudo contribui ao oferecer evidências locais sobre um agravo ainda pouco descrito entre bombeiros militares da região Norte.

Do ponto de vista sociodemográfico, observou-se predominância masculina e maior concentração de participantes com 40 anos ou mais, padrão semelhante ao descrito em levantamentos nacionais e internacionais^(20,21,22)^. Esse perfil pode refletir tanto a composição histórica das corporações quanto um possível envelhecimento da força de trabalho, fenômeno já documentado em serviços de emergência de outros países^(23)^. A maior participação feminina em comparação a estudos anteriores na Amazônia sugere mudanças graduais no perfil de ingresso, embora ainda em proporção reduzida.

No âmbito laboral, predominou o perfil de praças em funções operacionais, padrão compatível com corporações de bombeiros de diferentes países e com caracterizações da profissão no contexto brasileiro^(24)^. As jornadas caracterizadas por regime de turnos e realização frequente de horas extras podem estar relacionadas a maior exaustão física e mental, especialmente quando os períodos de descanso são insuficientes, conforme descrito em outros contextos ocupacionais^(25,26,27)^. Embora o delineamento transversal não permita estabelecer direção ou causalidade, tais condições configuram um ambiente de trabalho potencialmente propício ao acúmulo de demandas físicas e psicossociais.

A elevada frequência de sintomas osteomusculares, especialmente em regiões como coluna lombar, ombros, parte superior das costas, pescoço e joelhos, é coerente com o perfil de exigência física inerente à atividade de bombeiro militar^(26,27,28)^. Esses padrões podem ser parcialmente explicados por mecanismos biomecânicos amplamente descritos na literatura, como levantamento e transporte de cargas, deslocamentos rápidos, posturas forçadas, uso prolongado de equipamentos de proteção individual e movimentação de vítimas em ambientes instáveis. No contexto amazônico, fatores adicionais — como operações fluviais, longas distâncias de deslocamento, calor extremo e limitações estruturais — podem intensificar a demanda física e reduzir o tempo efetivo de recuperação entre turnos e, desse modo, contribuir para maior persistência dos sintomas.

A associação entre severidade dos DORT e lotação atual, embora de pequena magnitude, sugere que diferenças organizacionais e operacionais entre unidades podem influenciar a distribuição dos sintomas. As medianas mais elevadas observadas no COBOM e no 1.º- BI indicam que determinados contextos de trabalho podem estar relacionados a maior carga de sintomas, sem que seja possível inferir causalidade. Por outro lado, as associações desprezíveis ou fracas com sexo, nível hierárquico e idade sugerem que, nesta amostra, a severidade dos sintomas esteve menos relacionada a características individuais e mais vinculada ao contexto ocupacional e aos componentes clínico-funcionais captados pelo instrumento, achado que encontra respaldo em parte da literatura^(29,30)^.

As correlações elevadas entre o índice de severidade e variáveis como dor nos últimos 12 meses, dor nos últimos 7 dias, impedimento de atividades e procura por atendimento devem ser interpretadas como expressão da consistência interna do instrumento, e não como associações independentes. Esses indicadores integram a própria composição do índice, de modo que a magnitude observada reflete coerência estrutural do constructo, e não relações causais ou preditivas entre variáveis distintas.

Este estudo apresenta limitações que devem ser consideradas. A amostra por conveniência limita a generalização dos achados para toda a corporação. O uso de autorrelato pode introduzir vieses de memória ou percepção, especialmente em relação à intensidade e à duração dos sintomas. Além disso, embora tenham sido exploradas associações bivariadas, análises multivariadas poderiam oferecer estimativas ajustadas mais robustas com redução dos potenciais de confundimentos. Por fim, o delineamento transversal impede estabelecer temporalidade ou inferir causalidade entre exposições ocupacionais e severidade dos sintomas.

Adicionalmente, embora a aplicação de modelos multiva­riados — como a regressão de Poisson com variância robusta — pudesse fornecer razões de prevalência ajustadas, optou-se por restringir a análise ao plano bivariado, em coerência com o delineamento descritivo-exploratório e a amostragem por conveniência adotados. Tal escolha visa preservar a transparência inferencial e evitar a atribuição de precisão estatística incompatível com o desenho do estudo. Os achados aqui apresentados devem, portanto, ser interpretados como indicativos de associação, e não como estimativas de efeito ajustado generalizáveis à população de bombeiros militares.

Apesar dessas limitações, o estudo oferece evidências relevantes para a Enfermagem do Trabalho, ao caracterizar a prevalência e a severidade dos DORT em um contexto regional pouco explorado, o que reforça a necessidade de estratégias de vigilância em saúde, intervenções ergonômicas e ações organizacionais sensíveis às especificidades das diferentes lotações. Recomenda-se que investigações futuras, com delineamento longitudinal ou amostragem probabilística, apliquem modelos multivariados — como a regressão de Poisson com variância robusta — para estimar razões de prevalência ajustadas e explorar interações entre carga de trabalho, tempo de serviço e prática de atividade física, de modo a subsidiar programas preventivos baseados em evidências.

## CONCLUSÃO

A prevalência e a severidade dos distúrbios osteomusculares relacionados ao trabalho entre bombeiros militares de Manaus revelam uma carga expressiva de sintomas, especialmente em regiões corporais críticas para o desempenho operacional. As diferenças observadas entre lotações sugerem que características organizacionais e demandas específicas de cada unidade podem influenciar a distribuição dos sintomas, ainda que sem possibilidade de estabelecer causalidade.

Os achados reforçam a necessidade de ações sistemáticas de vigilância em saúde, intervenções ergonômicas e estratégias organizacionais que considerem as particularidades do contexto amazônico. Para a Enfermagem do Trabalho, os resultados oferecem subsídios para o planejamento de práticas preventivas, educativas e assistenciais voltadas à redução da carga osteomuscular e à promoção da saúde funcional desses profissionais. Estudos futuros com delineamentos analíticos e modelos ajustados poderão aprofundar a compreensão dos fatores associados aos DORT nessa população.

## Data Availability

Todo o conjunto de dados que dá suporte aos resultados deste estudo está disponível mediante solicitação ao autor correspondente.

# Translation: Prevalencia y gravedad de los trastornos musculoesqueléticos relacionados con el trabajo entre los bomberos militares de Manaos:un estudio transversal*

**DOI:** 10.1590/1980-220X-REEUSP-2025-0205es

**Authors:** Clodoaldo da Silva Almeida, Ana Lúcia Siqueira Costa Calache, David Lopes, Ednário Barbosa de Mendonça

**Affiliations:** 1 Universidade de São Paulo, Escola de Enfermagem, Programa Saúde do Adulto, São Paulo, SP, Brasil.; 2 Universidade Federal do Amazonas, Escola de Enfermagem de Manaus, Manaus, AM, Brasil.; 3 Universidade Federal de Campina Grande, Campina Grande, PB, Brasil.

**Keywords:** Trastornos de Traumas Acumulados, Bomberos, Salud Laboral, Ergonomía, Enfermería

## Abstract

**Objetivo:**

Evaluar la prevalencia y la gravedad de los trastornos musculoesqueléticos relacionados con el trabajo (TMET) en los bomberos militares de Manaos.

**Método:**

Estudio transversal con 272 bomberos en servicio activo (79,3 % del personal del Comando de Bomberos de la Capital/CBMAM). Se utilizaron un cuestionario sociodemográfico y laboral, así como el Cuestionario Nórdico de Síntomas Osteomusculares (QNSO, versión brasileña). La recolección de datos se realizó a través de REDCap (marzo de 2024). Los análisis se realizaron en RStudio, con pruebas no paramétricas (Mann-Whitney, Kruskal-Wallis, Spearman; α = 5 %) y medidas de efecto (IC 95 %).

**Resultados:**

Predominaron los hombres (76,8 %), con una edad promedio de 42,5 años, personal de campo (79,8 %) y actividades operativas (84,2 %). Se observó una elevada exposición laboral (turnos: 74,6 %; horas extras: 93,0 %) y la práctica regular de actividad física (87,9 %). La región lumbar presentó las mayores prevalencias de dolor, ausentismo y búsqueda de atención médica. La gravedad de los trastornos musculoesqueléticos relacionados con el trabajo (TMET) varió entre las unidades (p = 0,010; η^2^ = 0,045), con valores más elevados en el COBOM y en el 1.º BI. Las correlaciones entre el índice de gravedad y el dolor en 12 meses, el dolor en 7 días, la incapacidad y la búsqueda de atención médica reflejaron la consistencia interna del instrumento.

**Conclusión:**

Los bomberos militares presentaron una elevada prevalencia y gravedad de los trastornos musculoesqueléticos relacionados con el trabajo, con variaciones entre las unidades. Los hallazgos respaldan medidas de vigilancia en salud, ergonomía y organización del trabajo, especialmente en el ámbito de la enfermería del trabajo.

## INTRODUCCIÓN

La actividad del bombero militar es reconocida como una de las ocupaciones más exigentes desde el punto de vista físico y psicosocial, y se caracteriza por la exposición simultánea a riesgos físicos, químicos, biológicos, ergonómicos y organizacionales inherentes a las operaciones de rescate, combate de incendios y atención prehospitalaria^(1,2,3)^. Este conjunto de exigencias sitúa a esta categoría entre los grupos ocupacionales con mayor vulnerabilidad a los trastornos musculoesqueléticos relacionados con el trabajo (TMRT), afecciones que figuran entre las principales causas de baja laboral, incapacidad temporal y jubilación anticipada en las fuerzas armadas de todo el mundo^(4,5)^.

Estudios nacionales e internacionales han demostrado altas prevalencias de TMRT entre los bomberos, con tasas que entre el 50 % y el 80 % en períodos de 12 meses. Las zonas más afectadas comprenden la columna lumbar, los hombros, las rodillas y el cuello^(4,5,6,7)^. Estos patrones reflejan las exigencias biomecánicas de la actividad, entre las que se incluyen levantar y transportar cargas, adoptar posturas forzadas, el uso prolongado de equipo de protección personal y el traslado de víctimas^(6,8,9)^. Además, factores como la antigüedad en el servicio, el régimen de turnos, las jornadas prolongadas, la práctica insuficiente de actividad física y las características organizativas del trabajo se han asociado con la aparición y la gravedad de los TMRT, con impactos documentados sobre la capacidad funcional, el ausentismo y la calidad de vida^(2,3,7,9)^. En resumen, la literatura demuestra que los bomberos constituyen una población con una elevada carga de síntomas musculoesqueléticos, influenciada por exigencias físicas intensas y por condiciones organizativas específicas.

A pesar de este conjunto de evidencia, algunas cuestiones siguen sin resolverse, como la caracterización de los TMRT en contextos regionales específicos, especialmente en los cuerpos de bomberos militares de la región amazónica. Estos cuerpos se enfrentan a particularidades operativas —grandes distancias logísticas, condiciones climáticas adversas, incidentes fluviales de gran volumen, entornos forestales inhóspitos y limitaciones estructurales de apoyo— que pueden modificar tanto la exposición ocupacional como el perfil de las afecciones musculoesqueléticas^(10,11)^. A esto se suma la escasez de estudios brasileños que relacionen la prevalencia, la gravedad y la distribución anatómica de los TMRT con aplicabilidad directa a la enfermería del trabajo, sobre todo en la región Norte^(12)^.

Esta brecha limita la planificación de acciones de vigilancia y prevención que tengan en cuenta las realidades institucionales y territoriales de estas corporaciones, y sigue sin saberse si el perfil de afecciones musculoesqueléticas observado en otras regiones se reproduce en este contexto, caracterizado por particularidades climáticas, logísticas y asistenciales. Ante este panorama, generar evidencia local constituye un paso necesario para respaldar estrategias de monitoreo, prevención y atención coherentes con las especificidades de la población atendida —ámbito de actuación directa de la enfermería del trabajo—. Así, el presente estudio tuvo como objetivo evaluar la prevalencia y la gravedad de los trastornos musculoesqueléticos relacionados con el trabajo en bomberos militares de Manaos.

## MÉTODO

### Tipo y Lugar del Estudio

Estudio observacional, transversal y analítico, realizado en marzo de 2024 en el Comando de Bomberos de la Capital (CBC) del Cuerpo de Bomberos Militares de Amazonas (CBMAM). El estudio se llevó a cabo siguiendo las directrices de la iniciativa *Strengthening the Reporting of Observational Studies in Epidemiology – STROBE* ^(13)^.

### Población y Muestra

En el estudio participaron 343 bomberos militares, seleccionados mediante un muestreo no probabilístico de conveniencia. El número final de participantes (n = 272) se debió a la viabilidad de acceso y a la participación voluntaria de los bomberos durante el período de Recolección de datos, lo que corresponde aproximadamente al 79,3 % del personal del CBC, como se muestra en la Figura 1.

**Figura 1:** Flujo de muestreo de los participantes del estudio – CBC/CBMAM, Manaos, AM, Brasil, 2024.

**Fuente:** Cuestionario de la investigación (2024).

Se incluyeron bomberos militares en servicio activo, destinados en Manaos, en el estado de Amazonas, en Brasil. Se excluyeron aquellos que, durante el período de Recolección de Datos, se encontraban de vacaciones, de licencia (por salud, por premio o especial), en capacitación, ausentes por cualquier motivo, inactivos, que abandonaron la encuesta o con respuestas incompletas.

### Cálculo de la Muestra

Al tratarse de un muestreo no probabilístico por conveniencia, no se realizó un cálculo formal de la muestra. El número final de participantes (n = 272) se debió a la viabilidad de acceder a los bomberos en servicio activo, lo que representa el 79,3 % del personal del CBC, valor considerado suficiente para los propósitos de la investigación. Cabe destacar que, debido a la elección de este diseño, los hallazgos no tienen como objetivo la inferencia poblacional, sino la descripción del perfil y la identificación de asociaciones en el grupo estudiado.

### Recolección de Datos

La recolección se llevó a cabo en marzo de 2024 a través de un enlace enviado por WhatsApp desde los batallones. Tras aceptar la invitación y firmar el formulario de consentimiento libre e informado (TCLI), los participantes respondieron a los instrumentos en el software *Research Electronic Data Capture (REDCap)*, de la *Universidad de Vanderbilt*, que garantizó la recolección y la gestión seguras de los datos en línea. El proceso incluyó cinco etapas: presentación del proyecto, pruebas preliminares, envío de invitaciones, Recolección y almacenamiento de los datos, y exportación final para el análisis estadístico, de conformidad con la Ley General de Protección de Datos (LGPD)^(14)^.

### Instrumentos Utilizados

Se utilizó el Cuestionario Nórdico de Síntomas Osteo­musculares (QNSO en la versión en portugués), en su versión validada para su uso en Brasil^(15)^, empleado para evaluar la prevalencia y la gravedad de los síntomas osteomusculares, denominados en este estudio Trastornos Osteomusculares Relacionados con el Trabajo (TMRT).

El QNSO asigna un índice de gravedad a cada región anatómica, que varía de 0 a 4, donde 0 indica ausencia de síntomas; 1, síntomas en los últimos 12 meses o en los 7 días anteriores; 2, síntomas en ambos períodos; 3, presencia de síntomas con baja laboral; y 4, baja laboral prolongada asociada a síntomas en ambos períodos. Además, el instrumento evalúa las repercusiones funcionales del dolor, incluyendo la imposibilidad de realizar actividades habituales, variable utilizada en los análisis de correlación.

El índice de gravedad se calculó a partir de las puntuaciones del QNSO para cada región anatómica (de 0 a 4), en el que los valores más altos indican una mayor persistencia de los síntomas y una mayor repercusión funcional. Para cada participante, se obtuvo el promedio de las puntuaciones regionales, lo que una medida estandarizada de 0 a 4, comparable entre individuos y grupos. Dado que el índice resulta de la combinación de variables clínicas —dolor, impedimento para realizar actividades y búsqueda de atención médica—, las correlaciones con estos mismos componentes reflejan su estructura interna, lo que reduce la independencia estadística de las asociaciones. Por lo tanto, dichas correlaciones deben interpretarse con cautela, ya que expresan la consistencia interna del instrumento^(15)^.

### Tratamiento y Análisis de los Datos

Los datos se exportaron en formato CSV, se procesaron en Excel y se analizaron en RStudio (versión 2024.12.1). Los análisis descriptivos incluyeron frecuencias, promedios, desviaciones estándar y medianas. El índice de gravedad se evaluó de acuerdo con su estructura ordinal, y se aplicaron pruebas estadísticas no paramétricas, como Mann-Whitney, Kruskal-Wallis y la correlación de Spearman, para verificar asociaciones significativas, con un nivel de significación del 5 %. Además de las pruebas de significación, se calcularon medidas del tamaño del efecto para estimar la magnitud de las asociaciones: el eta cuadrado (η^2^) para la prueba de Kruskal–Wallis, el delta de Cliff (δ) para comparaciones entre dos grupos (Mann–Whitney) y el coeficiente de Spearman (ρ) con un intervalo de confianza del 95 % (IC95 %) para las correlaciones. Este enfoque complementa el análisis estadístico, poniendo de manifiesto no solo la significación, sino también la relevancia práctica de los resultados.

Cabe destacar que la medida de efecto delta de Cliff (δ) se utiliza para comparar dos grupos; los valores cercanos a 0,1 indican un efecto pequeño, 0,3 un efecto mediano y, si son mayores que 0,5, un efecto grande. El eta cuadrado (η^2^) se utiliza en comparaciones entre tres o más grupos (Kruskal–Wallis), cuyos valores de referencia son 0,01 = pequeño, 0,06 = mediano y 0,14 = grande; además, la correlación de Spearman (ρ) evalúa correlaciones monótonas, cuyo resultado interpretativo va de 0,1 a 0,3 para correlaciones débiles, de 0,31 a 0,5 para correlaciones moderadas y por encima de 0,5 para correlaciones fuertes. No se llevó a cabo ningún procedimiento de imputación de datos faltantes, ya que los participantes con respuestas incompletas fueron excluidos previamente de la muestra analítica, de acuerdo con los criterios de exclusión del estudio.

El análisis estadístico incluyó procedimientos descriptivos e inferenciales, con la presentación de las variables categóricas en frecuencias absolutas y relativas, y de las variables continuas en medidas de tendencia central y dispersión, según la distribución observada. Para la comparación de medianas entre grupos, se utilizaron las pruebas de Mann-Whitney y Kruskal-Wallis, dada la naturaleza no paramétrica de la variable de gravedad. Las correlaciones entre el índice de gravedad y las demás variables se estimaron mediante el coeficiente de Spearman, interpretadas según la clasificación convencional de magnitud.

Se reconoce que parte de las variables correlacionadas con el índice de gravedad —como el dolor en los últimos 12 meses, el dolor en los últimos 7 días, la limitación de actividades y la búsqueda de atención médica— forman parte de la propia composición del índice y configuran una relación de naturaleza estructural y no independiente entre constructos distintos. Esta característica se tuvo en cuenta en la interpretación de los resultados, en la que se evitó realizar inferencias causales a partir de estas asociaciones. Para futuras investigaciones que pretendan estimar asociaciones independientes entre exposiciones ocupacionales y resultados musculoesqueléticos en diseños transversales, se recomienda el uso de modelos que estimen directamente la razón de prevalencia (RP), como la regresión de Poisson con varianza robusta o los modelos log-binomiales^(16)^, en consonancia con las recomendaciones de la epidemiología contemporánea para los resultados prevalenciales^(17)^ y con los debates metodológicos sobre la adecuación de los estimadores en este tipo de diseño^(18)^.

### Procedimientos Éticos

El proyecto fue aprobado por el Comité de Ética en Investigación con Seres Humanos de la Universidad Federal de Amazonas, mediante el dictamen n.º- 6.632.276, y forma parte de la tesis de doctorado del autor.

## RESULTADOS

### Perfil Sociodemográfico y Conductual

Entre los participantes predominó el sexo masculino, con mayor concentración en el grupo de edad de 40 años o más. En cuanto a los hábitos de vida, el 43,8 % informó consumo de bebidas alcohólicas, mientras que el tabaquismo se registró en el 4,3 % de la muestra.

### Perfil Laboral

La mayoría de los participantes (79,8 %) pertenecía al nivel jerárquico de personal de base y realizaba actividades operativas (84,2 %). En cuanto a la antigüedad, el 75,7 % tenía entre 1 y 10 años de servicio en el CBMAM, patrón que también se observó entre quienes desempeñaban funciones operativas (82,7 %). Entre quienes realizaban tareas administrativas, el 96,7 % tenía menos de 10 años de experiencia, lo que podría sugerir una alta rotación.

Las exigencias laborales resultaron ser intensas: el 74,6 % de los participantes trabajaba por turnos y el 93,0 % realizaba horas extras en actividades operativas. A pesar de ello, el 87,9 % mencionó la práctica regular de actividad física, posiblemente como estrategia para mantener la aptitud funcional. En cuanto a la dotación de personal, se destacaron el Grupo de Rescate y Atención Prehospitalaria (GRAPH) (25,0 %), el Servicio de Combate a Incendios en Aeródromo (SCI) (20,2 %) y el Batallón de Bomberos Especiales (BBE) (18,4 %), lo que pone de manifiesto diferentes contextos de exposición a riesgos ocupacionales.

### Prevalencia de Trastornos Musculoesqueléticos Relacionados con el Trabajo

El Cuestionario Nórdico de Síntomas Osteomusculares (QNSO en portugués) reveló una alta prevalencia de TMRT, en particular en la región lumbar, que presentó la mayor frecuencia de dolor en los últimos 12 meses (44,1 %; IC 95 %: 38,1–50,2), además de los porcentajes más altos de baja laboral (23,5 %; IC del 95 %: 18,6–29,0), de consultas médicas (22,1 %; IC del 95 %: 17,3–27,5) y de dolor en los últimos 7 días (24,3 %; IC del 95 %: 19,3–29,8). También se destacaron los hombros, con una prevalencia de dolor en los últimos 12 meses del 37,5 % (IC 95 %: 31,7–43,5), la parte superior de la espalda, con un 35,3 % (IC 95 %: 29,6–41,3), y las rodillas, con un 32,4 % (IC 95 %: 26,8–38,3). Estos hallazgos sugieren una importante repercusión clínica y funcional de los síntomas en el grupo estudiado, tal como se muestra en la Tabla 1.

**Tabla 1:** Prevalencia de trastornos musculoesqueléticos relacionados con el trabajo por región corporal y bloques de análisis, con intervalos de confianza del 95 % entre bomberos militares de Manaos – AM, Brasil, 2024.

Región corporal	Dolor 12m% (IC95%)	Licencia por 12m% (IC95%)	Consultas 12m% (IC95%)	Dolor 7d% (IC95%)
Codos	11,0% (7,6–15,4)	6,6% (4,0–10,3)	5,5% (3,1–8,9)	6,6% (4,0–10,3)
Rodillas	32,4% (26,8–38,3)	18,4% (14,0–23,5)	16,9% (12,7–21,9)	19,5% (14,9–24,7)
Hombros	37,5% (31,7–43,5)	15,8% (11,7–20,7)	16,9% (12,7–21,9)	20,2% (15,6–25,5)
Parte baja de la espalda (lumbar)	44,1% (38,1–50,2)	23,5% (18,6–29,0)	22,1% (17,3–27,5)	24,3% (19,3–29,8)
Parte alta de la espalda	35,3% (29,6–41,3)	14,3% (10,4–19,1)	16,2% (12,0–21,1)	17,6% (13,3–22,7)
Cuello	34,6% (28,9–40,5)	10,3% (7,0–14,5)	13,2% (9,4–17,8)	16,9% (12,7–21,9)
Muñecas/Manos	26,1% (21,0–31,7)	11,0% (7,6–15,4)	10,7% (7,3–15,0)	13,6% (9,8–18,3)
Caderas/Muslos	26,8% (21,7–32,5)	13,6% (9,8–18,3)	14,0% (10,1–18,7)	13,6% (9,8–18,3)
Tobillos/Pies	25,7% (20,6–31,4)	15,1% (11,0–19,9)	12,1% (8,5–16,6)	14,3% (10,4–19,1)

Fuente: Cuestionario de la investigación (2024).

### Índice de Gravedad de los Trastornos Musculoesqueléticos Relacionados con el Trabajo

La distribución del índice de gravedad señaló un predominio de las categorías de mayor gravedad, con una elevada proporción de casos asociados a síntomas graves, bajas laborales y bajas prolongadas, lo que indica una imposibilidad de realizar actividades con posibles repercusiones en el ausentismo y el presentismo. Por el contrario, los niveles leves o moderados fueron menos frecuentes. Este patrón refuerza la idea de que, cuando estaban presentes, los síntomas tendían a manifestarse con mayor repercusión funcional en el grupo estudiado.

### Asociación entre Variables Sociodemográficas, Laborales y el Índice de Gravedad de los TMRT Relacionados con el Trabajo

Para examinar la relación entre las variables sociodemográficas y laborales y el índice de gravedad de los TMRT, se aplicaron pruebas no paramétricas (Mann–Whitney y Kruskal–Wallis), además de la correlación de Spearman para la edad. Se utilizaron las medidas de efecto correspondientes para estimar la magnitud de las asociaciones.

### Análisis de Significación Estadística

El índice de gravedad de los TMRT presentó un patrón centrado en la mediana de 3,00, con una dispersión similar (IQR = 2,00) en la mayoría de las categorías. No se observaron diferencias relevantes entre sexo y grado/cargo, y las distribuciones presentaron cuartiles similares. Por el contrario, la dotación de personal actual mostró variaciones entre las unidades, con valores relativamente más elevados en el 1.º BI y en el COBOM, y una distribución más baja en el SCI y en el GRAPH (Tabla 2).

**Tabla 2:** Comparación del índice de gravedad de los trastornos musculoesqueléticos relacionados con el trabajo, según variables sociodemográficas y laborales entre bomberos militares de Manaos – AM, Brasil, 2024.

Variable	Categoría/batallón	n	Mediana	Q1	Q3	IQR	p
Sexo	Masculino	209	3,00	1,00	3,00	2,00	0,885^M^
	Femenino	63	3,00	1,00	3,00	2,00	
Lugar de servicio	BIFMA – Batallón de Incendios Forestales y Medio Ambiente	30	3,00	1,00	3,00	2,00	0,010* ^K^
	BBE – Batallón de Bomberos Especiales	50	3,00	1,00	3,00	2,00	
	1.º- BI – Primer Batallón de Incendios	49	3,00	2,00	4,00	2,00	
	SCI – Servicio de Combate a Incendios en Aeródromos	55	3,00	1,00	3,00	2,00	
	GRAPH – Agrupación de Rescate y Atención Prehospitalaria	68	3,00	1,00	3,00	2,00	
	COBOM – Centro de Operaciones de Bomberos Militares	20	3,00	2,00	4,00	2,00
Grado/cargo	Soldado	217	3,00	1,00	3,00	2,00	0,287^M^
	Oficial	55	3,00	1,00	3,00	2,00	

Fonte: Questionário da pesquisa (2024).

Leyenda: (*) Diferencia significativa, p < 0,05.

^M^Prueba de Mann–Whitney;

^K^Prueba de Kruskal–Wallis. Q1 = primer cuartil; Q3 = tercer cuartil; IQR = intervalo intercuartílico (Q3–Q1).

### Magnitud del Efecto y Relevancia Práctica

En cuanto a la magnitud de las asociaciones, se observaron efectos insignificantes para el sexo (δ = 0,03) y el grado militar o cargo (δ = 0,08), lo que refleja una diferencia práctica mínima o nula en la gravedad entre estos grupos. En cuanto a la dotación actual de personal, se observó un efecto pequeño (η^2^ = 0,045), y el análisis post hoc indicó una mayor gravedad en el COBOM en comparación con el GRAPH y el SCI. La edad presentó una correlación débil y no significativa con el índice de gravedad (ρ = 0,12; IC 95 %: −0,01 a 0,25), lo que sugiere una influencia limitada de este factor sobre el resultado estudiado (Tabla 3).

**Tabla 3:** Asociación entre variables sociodemográficas y laborales y el índice de gravedad de los trastornos musculoesqueléticos relacionados con el trabajo entre bomberos militares de Manaos – AM, Brasil, 2024.

Variable	Categoría/descriptor	Medida de efecto	Interpretación	Comparaciones post-hoc (test de Dunn, corrección Bonferroni)
**Sexo**	Masculino/Feminino	δ = 0,03	Efecto insignificante	—
**Lugar de servicio**	1.º- BI – Batallón de Incendio; BIFMA – Incendio Forestal y Medio Ambiente; BBE – Bomberos Especiales; SCI – Combate a Incendio en Aeródromo; GRAPH – y Atención Prehospitalaria; COBOM – Centro de Operaciones Bombero Militar	η^2^ = 0,045	Efecto pequeño	COBOM > GRAPH, SCI (p < 0,05)
**Grado/cargo**	Soldado/oficial	δ = 0,08	Efecto insignificante	—
**Edad (Correlación de Spearman)**	Edad × Índice de Severidad	ρ = 0,12 (IC95%: −0,01–0,25)	Correlación débil, no significativa	—

Fuente: Cuestionario de la investigación (2024).

Leyenda: η^2^ = medida del efecto para la prueba de Kruskal–Wallis; δ = delta de Cliff para la prueba de Mann–Whitney; ρ = coeficiente de correlación de Spearman con un intervalo de confianza del 95 % (IC95 %).

### Consistencia Interna del Índice de Gravedad de los Trastornos Musculoesqueléticos Relacionados con el Trabajo

Para verificar la consistencia interna del índice de gravedad, se examinó la magnitud de la asociación entre el puntaje compuesto y los indicadores clínicos y funcionales que conforman su estructura. Los coeficientes de Spearman fueron elevados para el dolor en los últimos 12 meses (ρ = 0,896; IC 95 %: 0,870–0,917), el dolor en los últimos 7 días (ρ = 0,775; IC 95 %: 0,723–0,819), la limitación en las actividades habituales (ρ = 0,767; IC 95 %: 0,713–0,812) y la búsqueda de atención médica (ρ = 0,695; IC 95 %: 0,628–0,752), con p < 0,001 en todas las pruebas (Tabla 4).

**Tabla 4:** Coeficientes de consistencia interna entre los componentes del Cuestionario Nórdico de Síntomas Osteomusculares (QNSO) y el índice de gravedad de los TMRT, entre bomberos militares de Manaos – AM, Brasil, 2024.

Variable	Test	Medida de efecto (*)	Valor	p-valor	Interpretación	IC95%
Dolor en los últimos 12 meses × índice de gravedad	Spearman	ρ	0,896	<0,001	consistencia interna elevada	0,870 a 0,917
Dolor en los últimos 7 días × índice de gravedad			0,775	<0,001	consistencia interna elevada	0,723 a 0,819
Limitación de actividades × índice de gravedad			0,767	<0,001	consistencia interna elevada	0,713 a 0,812
Búsqueda de atención médica × índice de gravedad			0,695	<0,001	consistencia interna elevada	0,628 a 0,752

Fuente: Cuestionario de investigación (2024).

Leyenda: ρ = coeficiente de correlación de Spearman; IC95% = intervalo de confianza del 95%. Nota: Los coeficientes presentados reflejan la coherencia estructural entre los componentes del instrumento y la puntuación sintética (índice de gravedad), y no constituyen asociaciones independientes, dada la inclusión de las mismas variables en la composición del índice.

Estos resultados no deben interpretarse como asociaciones independientes, sino como una expresión de la consistencia interna del constructo, ya que los indicadores analizados forman parte de la propia composición del índice. La magnitud observada confirma que la puntuación sintetiza, de manera coherente, las dimensiones: temporal (presencia de síntomas en diferentes intervalos), funcional (limitación de las actividades) y asistencial (búsqueda de atención médica) propuestas por el QNSO, lo que refuerza su idoneidad como medida resumida de la gravedad de los TMRT en la presente muestra. La interpretación de estos coeficientes como evidencia de una asociación causal o predictiva entre variables clínicas y la gravedad sería, por lo tanto, metodológicamente inadecuada.

## DISCUSIÓN

Los hallazgos de este estudio se relacionan con la realidad operativa de los bomberos militares de la región amazónica, caracterizada por exigencias físicas intensas, condiciones ambientales adversas —entre las que se incluye la exposición a contaminantes atmosféricos provenientes de las quemas— y desafíos logísticos que marcan el día a día del trabajo^(19)^. Aunque estas características no se han medido directamente, conforman el entorno laboral en el que se insertan los participantes y ayudan a contextualizar la elevada carga de síntomas musculoesqueléticos observada. En este sentido, el estudio contribuye al ofrecer evidencia local sobre una afección aún poco descrita entre los bomberos militares de la región Norte.

Desde el punto de vista sociodemográfico, se observó un predominio masculino y una mayor concentración de participantes de 40 años o más, un patrón similar al descrito en estudios nacionales e internacionales^(20,21,22)^. Este perfil puede reflejar tanto la composición histórica de los cuerpos de bomberos como un posible envejecimiento de la fuerza laboral, fenómeno ya documentado en los servicios de emergencia de otros países^(23)^. La mayor participación femenina en comparación con estudios anteriores en la Amazonía sugiere cambios graduales en el perfil de ingreso, aunque aún en una proporción reducida.

En el ámbito laboral, predominó el perfil de personal de base en funciones operativas, un patrón compatible con los cuerpos de bomberos de diferentes países y con las caracterizaciones de la profesión en el contexto brasileño^(24)^. Las jornadas laborales caracterizadas por un régimen de turnos y la realización frecuente de horas extras pueden estar relacionadas con un mayor agotamiento físico y mental, especialmente cuando los períodos de descanso son insuficientes, tal como se ha descrito en otros contextos laborales^(25,26,27)^. Aunque el diseño transversal no permite establecer una dirección o causalidad, tales condiciones configuran un ambiente de trabajo potencialmente propicio para la acumulación de exigencias físicas y psicosociales.

La elevada frecuencia de síntomas musculoesqueléticos, especialmente en regiones como la columna lumbar, los hombros, la parte superior de la espalda, el cuello y las rodillas, concuerda con el perfil de exigencia física inherente a la actividad de los bomberos militares^(26,27,28)^. Estos patrones pueden explicarse en parte por mecanismos biomecánicos ampliamente descritos en la literatura, como el levantamiento y transporte de cargas, los desplazamientos rápidos, las posturas forzadas, el uso prolongado de equipo de protección personal y el traslado de víctimas en entornos inestables. En el contexto amazónico, factores adicionale, como las operaciones fluviales, los desplazamientos de largas distancias, el calor extremo y las limitaciones estructurales, pueden intensificar la exigencia física y reducir el tiempo efectivo de recuperación entre turnos y, de ese modo, contribuir a una mayor persistencia de los síntomas.

La asociación entre la gravedad de los TMRT y la dotación de personal actual, aunque de pequeña magnitud, sugiere que las diferencias organizativas y operativas entre las unidades pueden influir en la distribución de los síntomas. Las medianas más elevadas observadas en el COBOM y en el 1.º BI indican que ciertos contextos laborales pueden estar relacionados con una mayor carga de síntomas, sin que sea posible inferir una relación causal. Por otro lado, las asociaciones insignificantes o débiles con el sexo, el nivel jerárquico y la edad sugieren que, en esta muestra, la gravedad de los síntomas estuvo menos relacionada con características individuales y más vinculada al contexto ocupacional y a los componentes clínico-funcionales captados por el instrumento, hallazgo que encuentra respaldo en parte de la literatura^(29,30)^.

Las elevadas correlaciones entre el índice de gravedad y variables como el dolor en los últimos 12 meses, el dolor en los últimos 7 días, la limitación de actividades y la búsqueda de atención médica deben interpretarse como una expresión de la consistencia interna del instrumento, y no como asociaciones independientes. Estos indicadores forman parte de la propia composición del índice, de modo que la magnitud observada refleja la coherencia estructural del constructo, y no relaciones causales o predictivas entre variables distintas.

Este estudio presenta limitaciones que deben tenerse en cuenta. La muestra de conveniencia limita la generalización de los hallazgos a toda la corporación. El uso de la autoinformación puede introducir sesgos de memoria o percepción, especialmente en relación con la intensidad y la duración de los síntomas. Además, aunque se han explorado asociaciones bivariadas, los análisis multivariados podrían ofrecer estimaciones ajustadas más robustas al reducir los posibles factores de confusión. Por último, el diseño transversal impide establecer la temporalidad o inferir causalidad entre las exposiciones ocupacionales y la gravedad de los síntomas.

Asimismo, aunque la aplicación de modelos multivariados — como la regresión de Poisson con varianza robusta— podría proporcionar razones de prevalencia ajustadas, se optó por restringir el análisis al plano bivariado, en consonancia con el diseño descriptivo-exploratorio y el muestreo por conveniencia adoptados. Esta elección tiene como objetivo preservar la transparencia inferencial y evitar atribuir una precisión estadística incompatible con el diseño del estudio. Por lo tanto, los hallazgos aquí presentados deben interpretarse como indicativos de una asociación, y no como estimaciones de efecto ajustadas generalizables a la población de bomberos militares.

A pesar de estas limitaciones, el estudio ofrece evidencia relevante para la enfermería del trabajo, al caracterizar la prevalencia y la gravedad de los TMRT en un contexto regional poco explorado, lo que refuerza la necesidad de estrategias de vigilancia en salud, intervenciones ergonómicas y medidas organizacionales que tengan en cuenta las particularidades de las diferentes unidades. Se recomienda que las investigaciones futuras, con un diseño longitudinal o un muestreo probabilístico, apliquen modelos multivariados —como la regresión de Poisson con varianza robusta— para estimar razones de prevalencia ajustadas y explorar las interacciones entre la carga de trabajo, el tiempo de servicio y la práctica de actividad física, con el fin de respaldar programas preventivos basados en la evidencia.

## CONCLUSIÓN

La prevalencia y la gravedad de los trastornos musculoesqueléticos relacionados con el trabajo entre los bomberos militares de Manaos revelan una carga significativa de síntomas, especialmente en regiones corporales críticas para el desempeño operativo. Las diferencias observadas entre las unidades sugieren que las características organizativas y las exigencias específicas de cada una pueden influir en la distribución de los síntomas, aunque sin posibilidad de establecer una causalidad.

Los hallazgos refuerzan la necesidad de medidas sistemáticas de vigilancia en salud, intervenciones ergonómicas y estrategias organizacionales que consideren las particularidades del contexto amazónico. Para la enfermería del trabajo, los resultados ofrecen fundamentos para la planificación de prácticas preventivas, educativas y asistenciales orientadas a la reducción de la carga musculoesquelética y a la promoción de la salud funcional de estos profesionales. Estudios futuros con diseños analíticos y modelos ajustados podrían profundizar la comprensión de los factores asociados a los TMRT en esta población.

## Data Availability

El conjunto de datos que respaldan las conclusiones de este estudio está disponible previa solicitud al autor correspondiente.
